# The *Anemonia viridis* Venom: Coupling Biochemical Purification and RNA-Seq for Translational Research

**DOI:** 10.3390/md16110407

**Published:** 2018-10-25

**Authors:** Aldo Nicosia, Alexander Mikov, Matteo Cammarata, Paolo Colombo, Yaroslav Andreev, Sergey Kozlov, Angela Cuttitta

**Affiliations:** 1 National Research Council-Institute for the Study of Anthropogenic Impacts and Sustainability in the Marine Environment (IAS-CNR), Laboratory of Molecular Ecology and Biotechnology, Capo Granitola, Via del mare, Campobello di Mazara (TP), 91021 Sicily, Italy; 2 Shemyakin-Ovchinnikov Institute of Bioorganic Chemistry, RAS, GSP-7, ul. Miklukho-Maklaya, 16/10, 117997 Moscow, Russia; mikov.alexander@gmail.com (A.M.); ay@land.ru (Y.A.); serg@ibch.ru (S.K.); 3 Department of Earth and Marine Sciences, University of Palermo, 90100 Palermo, Italy; matteo.cammarata@unipa.it; 4 Istituto di Biomedicina e di Immunologia Molecolare, Consiglio Nazionale delle Ricerche, Via Ugo La Malfa 153, 90146 Palermo, Italy; paolo.colombo@ibim.cnr.it; 5 Institute of Molecular Medicine, Ministry of Healthcare of the Russian Federation, Sechenov First Moscow State Medical University, 119991 Moscow, Russia

**Keywords:** transcriptomics, bio-prospecting, computational biology, neurotoxins

## Abstract

Blue biotechnologies implement marine bio-resources for addressing practical concerns. The isolation of biologically active molecules from marine animals is one of the main ways this field develops. Strikingly, cnidaria are considered as sustainable resources for this purpose, as they possess unique cells for attack and protection, producing an articulated cocktail of bioactive substances. The Mediterranean sea anemone *Anemonia viridis* has been studied extensively for years. In this short review, we summarize advances in bioprospecting of the *A. viridis* toxin arsenal. *A. viridis* RNA datasets and toxin data mining approaches are briefly described. Analysis reveals the major pool of neurotoxins of *A. viridis*, which are particularly active on sodium and potassium channels. This review therefore integrates progress in both RNA-Seq based and biochemical-based bioprospecting of *A. viridis* toxins for biotechnological exploitation.

## 1. Introduction

The exploitation of marine bio-resources aims to respond to critical needs of society supporting development in different fields of the blue biotechnologies which include drug discovery, bioremediation, biomaterials and aquafarming. Over the years, the screening of marine extracts for bioactive products has resulted in successful marine compounds screening and drug discovery, which has passed clinical trials [1,2]. Marine organisms are considered one of the largest reservoirs of natural molecules to be evaluated for drug activity, and among them, Cnidaria represent an ancient group of venomous animals specialized in toxins production and delivery [3].

Cnidaria are soft-bodied organisms which lack a traditional protection system such as as cuticle or exoskeleton, hemolymph and phagocytes, thus resulting in it being continuously exposed to diverse pathogens. Their defense systems are of growing interest, as they likely contain molecules able to counteract pathogen infection. Sea anemones (Actinaria, Cnidaria) are ancient sessile predators [4] whose survival mainly relies on their venom production [5] and nematocysts, specialized cells for stinging and delivery of venoms, are usually used to immobilize prey or dissuade predators.

Analyses of the venoms of many sea anemone species has uncovered a variegate arsenal of low molecular weight molecules [6]. Because the capture and killing of prey as well as mechanisms of defense and protection in sea anemones are closely related to toxin production, the presence of multiple toxin variants could provide some benefit [7].

The sea anemone *A. viridis*, previously known as *Anemonia sulcata*, is an extensively studied Mediterranean species [8,9,10,11,12,13,14,15]. More than 20 polypeptide toxins of different structures and functions have been isolated from crude extracts of this species. They include potassium channel blockers, such as kalicludines, kaliseptine, blood depressing substance (BDS), neurotoxins blocking sodium channels, and Kunitz-type inhibitors of proteolytic enzymes [16]. Rapid advances in DNA sequencing technologies resulted in growing bio-prospecting efforts for the screening and identification of novel sea anemone toxin sequences [17,18,19], thus enabling *A. viridis* to be considered as a reliable source of bioactive molecules.

## 2. *A. viridis* RNA Datasets and Data Mining

Large-scale datasets and transcriptome collections freely available on public databases represent priceless resources for the mining of gene products aiming at biotechnological exploitation.

In silico bioprospecting based on “big data” derived from RNA deep sequencing provided a boost in the screening of candidates for biomedicine and biotechnology applications, thus complementing canonical bio-guided fractionation procedures.

In short, projects of massively parallel sequencing have produced several millions of reads per run, and thus require extended computing resources for storage and data processing. Based on annotation procedures, homologs detection, motifs scan, and gene ontology analysis, different strategies are usually exploited for the identification of candidate gene products.

To date, 17 transcriptome datasets have been identified at NCBI using as query «*Anemonia viridis*» or «*Anemonia sulcata*» (Table 1)*.* The majority of retrieved datasets were generated using Illumina HiSeq or SOLiD platforms; while the transcriptome of the symbiotic sea anemone *A. viridis*, at first was analyzed in depth using expressed sequence tags (EST) libraries combined with traditional Sanger sequencing [20], which retrieved 39,939 high quality ESTs, assembled into 14,504 unique sequences.

More recently, tissue-specific paired-end libraries were generated and sequenced on Illumina HiSeq platforms in order to characterize the venom composition among different anatomical districts of the sea anemone [19]. Additionally, in-depth small RNA libraries were produced and sequenced based on SOLiD technology enabling both the identification of novel miRNA and the analyses of differential miRNA expression under pH gradients [21].

For successful in silico bio-prospecting of toxin-like candidates, different approaches have been implemented. They exploit advances on homologues identification on the basis of hidden Markov models (HMMs) libraries built on a curated seed alignment of Pfam domains and motif recognition of specific key amino acid residues distribution which are characteristic of the different toxin types [17,18]. In particular, for candidate toxins of *A. viridis*, single residue distribution analysis (SRDA) was successfully used for the determination of a motif array based on alignment of curated cnidarian toxins, which takes into account the distribution pattern of Cys residues [17].

The RNA-Seq libraries were usually interrogated using these Cys-motifs (Cys-patterns), and translated sequences that satisfy each query were selected.

**Figure 1:**
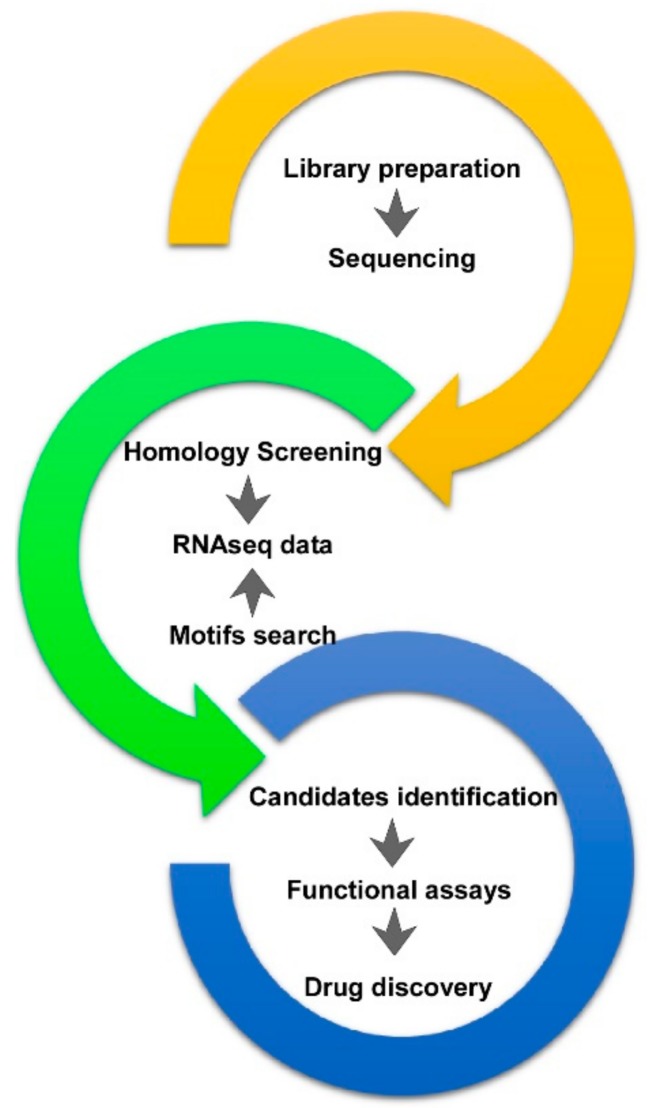
Pipeline for in silico bio-prospecting and candidate toxins identification. This includes library preparation, RNA deep sequencing, data analyses by motif and/or homology screening, and recovery of matching sequences, expression and subsequent functional testing.

Thus, as a pipeline for in silico bio-prospecting, the workflow leading to candidate toxins identification is reported in Figure 1.

Similar to NGS platforms, in recent years, the developments in mass spectrometry, public repository for proteomics data (such as PRoteomics IDEntifications –PRIDE– [22] PeptideAtlas-PASSEL– [23] ProteomicsDB [24], Mass Spectrometry Interactive Virtual Environment-MassIVE–) and algorithms for integrated searches have provided useful tools for proteogenomics applications [25,26] as venomics [27]. However, to date, no proteomics and mass spectrometry data have been found for *A. viridis*; while similar approaches have been successfully used in deep venomics of other organisms ([27] and references therein). The integration of RNA-seq data into the proteomics for validation of *A. viridis* candidate toxins against mass spectrometric data will establish a standardized OMICS approach for deep bio-discovery.

## 3. The Multifaceted Molecular Arsenal of Sea Anemone *A. viridis*

Sea anemones are known as a source of peptide toxins, acting mainly on ion channels as blockers or modulators, particularly in excitable cells. It has been reported that venomous animals are able to produce toxins that act on different molecular targets on the same physiological system. Toxins mainly act by altering the activity of neurons by either blocking/changing the kinetics of Na^+^ channels [28,29] or by blocking of neuronal K^+^ channels [30]. However, the chemical arsenal is not limited to modulators of excitability in prey; it also includes molecules altering membrane permeability or inducing the formation of pores in the membrane. Therefore, analyses of sea anemone venoms revealed that they contain a diversity of biologically active proteins and peptides which have not been fully explored until the exploiting of transcriptomic and/or proteomic procedures. Herein, data reporting canonical bioactive compounds in isolation were combined with in silico bio-prospecting on *A. viridis* RNA datasets to provide a comprehensive synopsis of a multifaceted molecular arsenal of the well-studied sea anemone *A. viridis*. To avoid confusion, toxins and other proteins herein collected were named accordingly to nomenclature provided in the databases or in previous reports.

### 3.1. Sodium Channel Peptide Toxins

Voltage-gated sodium channels (Na_V_s) display an important role in neuron signal transduction events, as they are responsible for the instauration of action potential and conduction of electric impulses. Reasonably, Na_V_s represent eligible targets for sea anemone toxins. Initially, sea anemone toxins were discovered in venoms composition utilizing classical biochemical and bio-guided fractionation procedures. This generally applies for all the sources of bioactive compound species, as well as for *A. viridis*, specifically. During the years, the screening of different cDNA and genome libraries [9], as well as a bio-prospecting approach on the EST and RNA-Seq collection of the Mediterranean sea anemone allowed for the identification of a novel class of candidate toxins and confirmed the existence of already known sodium channel toxins.

Historically, the sea anemone Na_V_-toxins have been grouped into three types on the basis of amino acid sequences, length and S-S bonds arrangement (type I, II and III).

The *A. viridis* Type I-II toxins bind specifically to the Na_V_s of excitable tissue, prolonging the open state of the channels during the depolarization process. Thus, a delay in the inactivation process is usually achieved when applying these toxins on the soma membrane of crustacean neurons [31,32]. Because of this specialized activity on the Na_V_s channels, these toxins [33] may be used as reliable tools for physiological investigations focused on Na_V_s function.

Type I and II are represented by toxin molecules with 46–49 amino-acid residues. They were preferentially isolated from the organisms belonging to the families Actinidae and Stichodactylidae. The mature peptides inside each of these groups show similar structural features. Type I and II molecules share the same Cys-framework (I–V, II–IV, III–VI). More precisely, Type I disulphide connection pattern may be defined as (4–44, 6–34, 27–45), and for Type II as (3–17, 4–11, 6–22)—but the general framework is the same (I–V, II–IV, III–VI). Both molecules with confirmed experimental evidence at the protein level and those that were deduced only from transcriptomics data are included in Type I and Type II groups.

According to classification of Cys-distribution patterns in sea anemone venom toxins proposed by Mikov and Kozlov [34], type I *A. viridis* Na_V_ toxins belong to structural group 1a because they fall into the following pattern: C1C##C6C–X_1_–CC#, where X_1_ = 6–9 aa, ## = more than 9 aa, # = 1–9 aa. Moreover, type II *A. viridis* Na_V_ toxins belong to structural group 1a because they fall into the pattern: CC1C–X_1_–C5C4C#, where X_1_ = 2 or 4 or 5 aa, # = 1–9 aa.

In the light of biotechnological exploitations, it is paramount to describe toxins with confirmed experimental evidence at the protein level (especially ones with established activity), because similar peptides are usually being found by inference from homology with them.

*Type I Nav toxins*. The following three toxins were found to be present in venom at the protein level:

(1) neurotoxin 1 (δ-actitoxin-Avd1a; ATX Ia);
(2) neurotoxin 2 (δ-actitoxin-Avd1c; ATX II);
(3) neurotoxin 5 (δ-actitoxin-Avd1d; ATX-V).

Neurotoxin 1 (ATX Ia; UniProt ID: P01533) amino-acid sequence was published back in 1978 [35]. It was shown then that neurotoxin 1 has an effect on Na^+^ currents inactivation, but it does not affect K^+^ and Ca^2+^ currents in crustacean neurons with concentrations up to 5 μM [35]. In the presence of ATX Ia, inactivation of crustacean neuron Na^+^ currents was incomplete. Moreover, some parameters of activation were also affected by ATX Ia, for example, the negative resistance branch of the peak Na^+^ current-voltage relation had been shifted [35]. The secondary structure of neurotoxin 1 was determined using complete sequence-specific NMR-assignments [36]; it was shown that it comprises β-sheets consisting of four strands, and no evidence of helical structures was found. The three-dimensional structure of neurotoxin 1 in aqueous solution was determined by nuclear magnetic resonance [37]. Neurotoxin 1 has a well-defined molecular core formed by four-stranded β-sheet that is connected by two well-defined loops (defensin-like fold). The core is stabilized by three disulphide bridges (PDB ID: 1ATX). There is also an additional flexible loop made of 11 residues (8–18 aa).

Neurotoxin 2 (ATX II; UniProt ID: P0DL49) amino-acid sequence was established and published in 1976 [33], while disulphide bridges coordination pattern of this peptide was published later in 1978 [38]. However, the three-dimensional structure of ATX II has not been determined experimentally yet. First, physiological investigation of ATX II was described in the same paper as for ATX Ia [35]. In general, action of ATX II was reminiscent of that of ATX Ia—it inflicted selective Na^+^ current inactivation slowdown with no effect on K^+^ and Ca^2+^ currents and changed current-voltage relation for activation at the same time in crustacean neurons. Additionally, ATX II led to shortening of the action potential in crayfish neurons in experiments with repetitive stimulations [39].

Neurotoxin 1 was tested only on crayfish neurons. Neurotoxin 2, however, was subjected to more extensive investigations. ATX II was applied on α-subunits of hH1 (human heart subtype 1), rSkM1 (rat skeletal muscle subtype 1) and hSkM1 (human skeletal muscle) sodium channels expressed in the tsA101 line of human embryonic kidney cells to reveal broad pharmacological properties of this peptide [40]. Neurotoxin 2 revealed potent slowing of the inactivation phase of hH1 (IC_50_ 11 nM) as well as moderate slowing of the inactivation phase of rSkM1 (IC_50_ 51 nM) and hSkM1 (IC_50_ ~ 50 nM). There was no effect of ATX II on the activation of any of these sodium channels [40]. The example of studying pharmacological and physiological properties of neurotoxin 2 represents a good example of the urgency of testing novel toxins on a wide enlistment of channel types and subtypes as a possibility to reveal a real selectivity/range of actions of the particular toxin.

Neurotoxin 5 (ATX-V; UniProt ID: P01529) amino acid sequence was published in 1982 [40]; it was already known that this peptide is highly toxic for mammals. It was revealed that ATX-V delays inactivation of sodium currents and, therefore, strongly stimulates mammalian cardiac muscle contraction.

**Figure 2:**
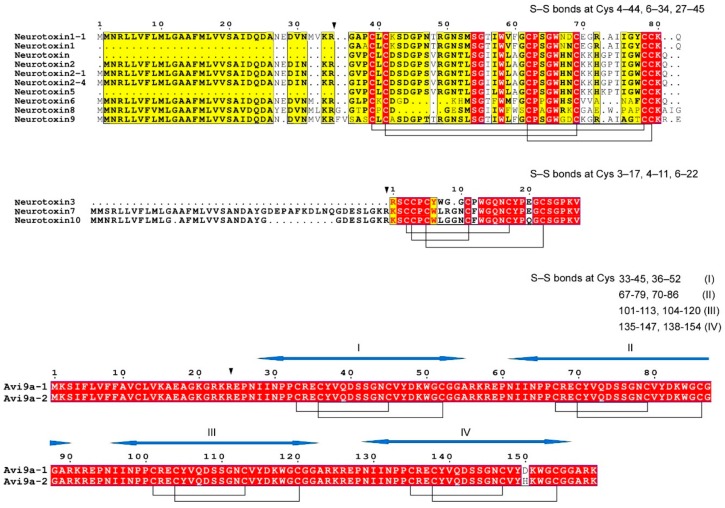
Multiple sequence alignment of the Na_V_s toxins in *A. viridis*. Based on S-S bonds arrangement, Nav toxins are reported as Type I on the top, Type II on the middle and Type III on the bottom. Alignment was performed with T-coffee tool [43]. Similar residues are written in bold characters and boxed in yellow, whereas conserved residues are in white bold characters and boxed in red. The sequence numbering on the top refers to the alignment. For each alignment, the pattern of Cys residues forming disulfide bridges is shown. Pro-peptide processing sites are pointed out by an inverted black triangle. The four motifs of the active toxins for Avi 9a-1 and Avi 9a-2 are indicated by the blue arrows.

*Type II Nav toxins*. Concerning Type II molecules, only 1 peptide—neurotoxin 3 (δ-actitoxin-Avd2a; ATX III; Av3; UniProt ID: P01535) was present in venom at protein level. Neurotoxin 7 and neurotoxin 10 sequences were deduced from transcriptomes. Interestingly, ATX III, isolated earlier from the sea anemone venom [41], was not retrieved neither from the dbESTs, nor from any RNA-Seq datasets available to date. However, such peptides as Neurotoxin 7 and Neurotoxin 10 showed analogous features and exhibited a pattern of disulfide bonds connectivity (3–17, 4–11, 6–22; pattern: I–V, II–IV, III–VI) which is characteristic for ATX III (Figure 2).

Initially, is was shown that neurotoxin 3 had a similar activity mode towards crayfish neuron currents as toxins of Type I (slowing down inactivation of Na^+^ channels, etc.) [30]. ATX III had no effect on K^+^ and Ca^2+^ currents when tested on concentrations up to 5 µM. ATX III had selective effects on the Na^+^ currents at the lowest tested concentration of 50 nM; the effect had the same nature as the action of ATX I and ATX II (influence on both inactivation and activation parameters). Moreover, it was shown that ATX III is toxic to crustaceans and is not toxic to mammals [35]. The structure-functional traits of neurotoxin 3 were studied in detail in the following comprehensive work by Moran et al. [42]. As previous data revealed, ATX III is highly toxic to crustaceans while being harmless to mice. Moran et al. suggested that ATX III might be selectively active on arthropods in general (not just crustaceans) [42]. The experiments demonstrated that Av3 is highly toxic to blowfly larvae (ED_50_ 2.65 ± 0.46 pmol/100 mg) and that it effectively competes with classical site-3 scorpion toxin LqhαIT on binding to cockroach neuronal membranes [42]. Moreover, Av3 inhibited the inactivation of the fly *Drosophila melanogaster* channel DmNav1, but did not influence the functioning of mammalian Na_V_ channels. Furthermore, action of ATX III was significantly intensified by receptor site-4 ligands, such as scorpion β-toxins [42]. Av3 presented itself as a quite unusual site-3 inhibitor of sodium channels: D1701R mutation in DmNav1 channel did not influence the action of Av3, while the actions of other site-3 ligands were abolished [42]; therefore, this toxin may possess a unique mode of interaction with DmNav1 and this needs to be studied in detail.

*Type III Na_V_ toxins*. Type III includes shorter polypeptides with 27–31 aa residues. These toxins were also found in the arsenal of *A. viridis*. Type III toxins of A. viridis are encoded into complex 159 amino acid-long precursor proteins named AnmTX Avi 9a-1 and AnmTX Avi 9a-2 (short names are Avi 9a-1 and Avi 9a-2). Arbitrarily, mature toxins coded by these long precursors are also named Avi 9a-1 and Avi 9a-2. As mature toxins, Avi 9a-1 and Avi 9a-2 are short peptides almost identical in their primary structure (the only difference is His-22 in Avi 9a-2, instead of Asp-22 in Avi 9a-1). Interestingly, precursor protein AnmTX Avi 9a-1 includes four copies of Avi 9a-1 toxin, while precursor protein AnmTX Avi 9a-2 includes three copies of Avi 9a-1 toxin and one copy of Avi 9a-2 toxin (see Figure 2). To the best of our knowledge, there are no works confirming evidence of short toxins Avi 9a-1 and Avi 9a-2 on protein level and there are no works on the activity of these toxins; there is only evidence on transcript level [17]. Mature Avi 9a-1 and Avi 9a-2 toxins are homologous to toxin π-AnmTX Ugr 9a-1 which is known as an inhibitor of human type 3 asid-sensing ion channels (hASIC3) [44]. Moreover, they are similar to Am I toxin from the sea anemone *Antheopsis maculata* in their primary structure [45]. The homology does not allow making any conclusions on Avi 9a-1 and Avi 9a-2 actual neuropharmacology, so solid experimental evidence is needed. Concerning Cys-distribution pattern, Type III *A. viridis* Na_V_ toxins belong to structural group 9a because they comply with the following pattern: C2C#C#C#, where # = 1–9 aa (according to Reference [33]).

### 3.2. Potassium Channel Peptide Toxins

Sea anemone K^+^ channel-blocking toxins have been grouped into different classes (type 1–4) based on the number of amino acid residues, disulfide bridge patterns and molecular structure [46,47]. The mature form of Type 1 and 2 toxins consists of about 40 and 58 amino acid residues, respectively; whereas three disulphide bridges stabilize the structures of these molecules. Type 1 peptides include ShK toxins from the sea anemone *Stichodactyla helianthus*. The ShK represents one of first K^+^ channel-blocking toxins from marine organisms which was structurally and functionally characterised because of specific activity on K_V_1.3, K_V_1.1 and K_V_3.2 [48,49] channels; while type 2 peptides, which include the kalicludines, are similar to Kunitz-type protease inhibitors.

*Type 1 K_V_ blockers*. *A. viridis* members of Type 1 family include kaliseptine (AsKS; κ-actitoxin-Avd6a; UniProt ID: Q9TWG1)—36 aa peptide, which has no sequence homology with kalicludines from *A. viridis* or with dendrotoxins from mamba snake venoms. Kaliseptine was isolated from *A. viridis* venom during the separation combined with tests of fractions on their ability to compete with ^125^I-dendrotoxin I (^125^I-DTX_I_) for binding its receptors, K^+^ channels, in rat brain membranes [50]. IC_50_ for inhibition of ^125^I-DTX_I_ binding by AsKS is 27 nM, while DTX_I_ itself competes with ^125^I-DTX_I_ much more actively than with IC_50_ 0.14 nM. Interestingly, AsKS does not compete with ^125^I-calcicludine for its binding to Ca^2+^ channels of the rat brain up to a concentration of 5 µM [50]. Electrophysiological experiments using different K_V_ channels expressed in Xenopus oocytes revealed that kaliseptine inhibits K_V_1.2 currents with IC_50_ 140 nM, while DTX_I_ had IC_50_ 2.1 nM [50]. Unlike kalicludines, kaliseptine has no homology with bovine pancreatic trypsin inhibitor and, therefore, does not inhibit trypsin even at very high concentrations.

**Figure 3:**
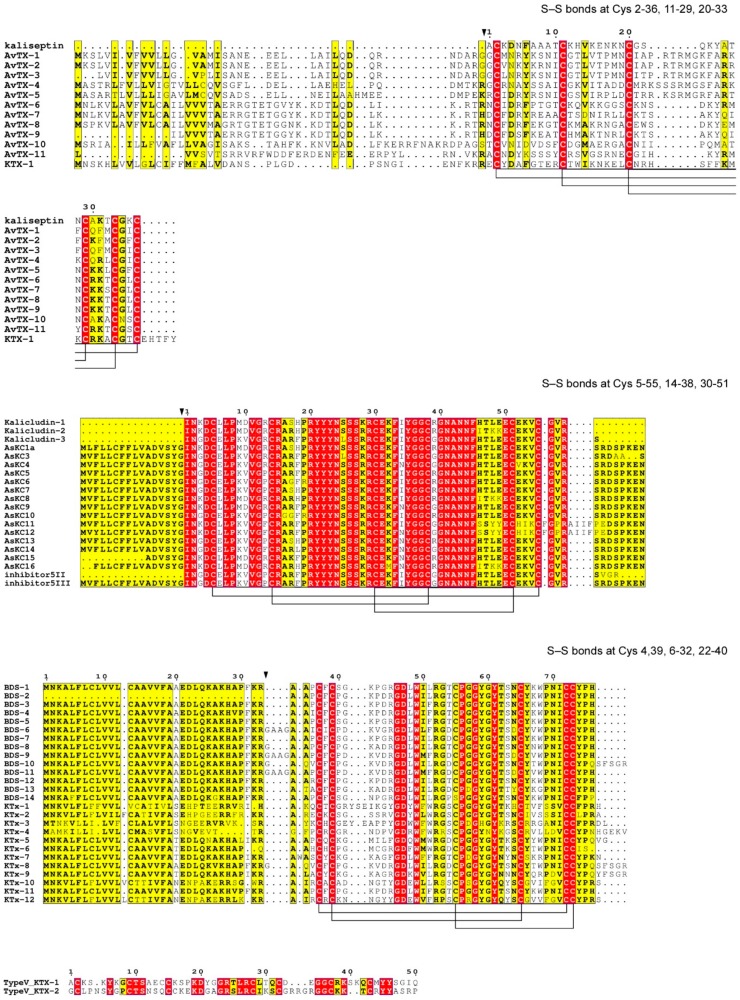
Multiple sequence alignment of the K_V_s toxins in *A. viridis*. Alignment was performed with the T-coffee tool [43]. Similar residues are written in bold characters and boxed in yellow, whereas conserved residues are in white bold characters and boxed in red. The sequence numbering on the top refers to the alignment. For each alignment, the pattern of Cys residues forming disulfide bridges is shown. Type 1, 2, 3 and 5 K_V_ blockers are reported; while no member of Type 4 has been identified to date. No S-S bonds and Cys pattern are defined for type V KTx because of the absence of any 3D structure experimentally determined to date.

Based on polypeptide length and exhibition of a specific pattern of S-S bonds (2–36, 11–29, 20–33; I–VI, II–IV, III–V), biochemical isolation and motif scan on RNA datasets retrieved the existence of a Type 1 K^+^ toxin subset including 12 peptide toxins with substantial homology to kaliseptine (AvTx1–11 [17] and KTX-1 [19] (Figure 3). It may be easily predicted by the SignalP algorithm [51] that after the proteolytic releases mature toxins, AvTx1–11 and KTX-1 consist of about 40 amino-acid residues. None of these molecules were tested for pharmacology properties, so whether these toxins possess K_V_-blocking or proteinase-inhibiting activity is an open issue for future investigations. Type 1 A. viridis K_V_ blockers belong to structural group 2a, because they conform with the following Cys-pattern: C8C–X_1_–C*C3C2C, where X_1_ = 8 or 4, and *—is for highly variable intervals (according to Reference [33]).

*Type 2 K_V_ blockers*. Several members of type 2 K^+^ channel-blocking toxins were also found in *A. viridis*. The complex array included 22 kalicludine variants (AsKCn), 2 proteinase inhibitors (5 II and 5 III) and 24 additional type 2 peptides (KTx2n) showing a molecular architecture similar to Kunitz-type protease inhibitors. Similar to kaliseptines, kalicludines are able to block K_V_ channels containing K_V_1.2 subunits [50]. Interestingly, in silico bio-prospecting also revealed the production of two non-canonical type V KTx toxins. The founder members of K_V_ type V toxins were recently isolated from the venom of the sea anemone *Bunodosoma caissarum* and showed a high affinity for different channels including K_V_1.1, K_V_1.2, K_V_1.3, K_V_1.6 and Shaker IR [52].

The initial pipeline for identification of three kalicludines (AsKC1–3) was the same as for kaliseptine [50]. Similar to kaliseptine, kalicludines are able to block rat brain K_V_ channels containing K_V_1.2 subunits [42]. The IC_50_ for inhibition of the K_V_1.2 current was 1.1 mM for AsKC2, 1.3 mM for AsKC3, and 2.8 mM for AsKC1 (compared to IC_50_ 2.1 nM for DTX_I_); so kalicludines are quite weak blockers of K_V_1.2 channels [50]. Similar to AsKS, kalicludines do not compete with ^125^I-calcicludine for its binding to Ca^2+^ channels of the rat brain up to a concentration of 5 µM. However, in contrast to AsKS, AsKCs exhibited proteinase-inhibiting properties, and inhibited trypsin with K_d_ below 30 nM; therefore, kalicludines are molecules with dual type of activity. Combination of trypsin inhibition and K_V_-blocking properties is very peculiar to toxins with Kunitz-type fold. So, despite the fact that 3D structures of kalicludines have not been determined yet, a combination of activity and Cys-distribution patterns may be proposed so that AsKCs possess a 3D structure similar to classical Kunitz-type molecules. Disulphide connection framework is CysI–CysVI, CysII–CysIV, CysIII–CysV and the structure consists of a combination of N-terminal 3_10_-helix, C-terminal α-helix and 3-stranded anti-parallel β-structure in the core [53,54,55,56]. Sequences of kalicludines AsKC1-3 have been extracted from EST databases, so their real presence in the venom needs to be validated by rigorous proteomics investigations and their biological activity is to be established in the future. Type 2 *A. viridis* K_V_ blockers belong to structural group 3a because they comply with the following pattern: C8C15C7C12C3C (classification [1]).

*Type 3 K_V_ blockers*. The Mediterranean sea anemone *A. viridis* also produces Type 3 toxins which are represented by BDS peptides (1–14) and 12 Type 3 KTx. Members of this class include peptides of various origins. For example, APETx1 (inhibits K_V_11.1, K_V_11.3 and blocks Na_V_1.2, Na_V_1.3, Na_V_1.4, Na_V_1.5, Na_V_1.6, Na_V_1.8 of mammals) and APETx2 (inhibits mammalian K_V_3.4, K_V_11.1, some Na_V_ and blocks ASIC3-containing trimers) were isolated from *Anthopleura elegantissima* venom [57,58,59]. Another example is Am-II from *Antheopsis maculata* which is of unknown pharmacology but is toxic to crabs [45]. As might be seen, Type 3 toxins may exhibit a lot of diversified properties besides being K_V_-blockers.

The initial members of this class from *A. viridis* venom—BDS-1 (UniProtID: P11494) and BDS-2 (UniProtID: P59084)—were originally described as blood pressure-reducing substances with antiviral activities [60]. These are 43 amino acid long, cysteine-rich polypeptides stabilized by three disulphide bonds. The peptides voltage-dependently inhibit K^+^ channels containing K_V_3 subunits such as K_V_3.1, K_V_3.2, and fast inactivating channel K_V_3.4 [61,62,63]. Additionally, it has been recently reported that BDS-1 also slows down inactivation of the human Na_V_1.7/SCN9A channels (and Na_V_1.7 in rat SCG neurons as well), and weakly inhibits Na_V_1.3 channels [64].

Type 3 K_V_ blockers belong to a Cys-distribution pattern named “1b” (classification [1]) which implies the following distribution scheme: C1C##C9C–X_1_–CC#, where X_1_ = 6–8, # = 1–9 aa, ## = more than 9 aa. Concerning *A. viridis* molecules, the spatial structure was resolved for BDS-1 by NMR [65] (concerning other species, 3D-structures were also determined for APETx1 [66] and APETx2 [67]). These 3D-structures are quite similar for BDS-1, APETx1 and APETx2—each of them may be described as a combination of the compact disulphide-stabilized nucleus consisting of a four-stranded β-sheet with N- and C-termini protruding from it (the so-called defensin-like fold, the same as for structural pattern 1a).

*Type 4 K_V_ blockers*. Toxins of this Type were not identified in the venom of *A. viridis* as well as in its transcriptomes. This may be both due to technical limitations and difficulties or simply because *A. viridis* does not produce Type 4 K_V_ blockers. This issue is to be resolved in future investigations.

*Non-canonical Type 5 KTx-like peptides*. Interestingly, in silico bioprospecting also revealed the production of two non-canonical Type V KTx-like peptides. The founder members of K_V_ Type 5 toxins were recently isolated from the venom of the sea anemone *B. caissarum* and showed a high affinity for different channels including K_V_1.1, K_V_1.2, K_V_1.3, K_V_1.6 and Shaker IR [52].

### 3.3. Other Candidate Toxins of A. viridis

In addition to the peptide toxins whose functions have been well characterized, several putative polypeptides were retrieved only by means of bio-prospecting on transcriptome datasets.

*Gigantoxin homologs*. This group includes gigantoxin 4 (U-AITX-Avd12a; UniProtID: P0DMY9) and gigantoxin 5 (U-AITX-Avd12b; UniProtID: P0DMZ0). They show significant similarity with gigantoxin Ι from the giant carpet sea anemone *Stichodactyla gigantea* [68]. Gigantoxin Ι (ω-stichotoxin-Sgt1a; UniProtID: Q76CA1) is known to exert a potent paralytic and weak lethal effect on crabs (PD_50_ is 215 µg/kg; LD_50_ is > 1000 µg/kg) [68]. Moreover, gigantoxin Ι is capable of binding to epidermal growth factor receptor (EGFR) and, therefore, induces morphological changes (rounding of the cells) and tyrosine phosphorylation of the EGFR in cells (was shown using epidermoid carcinoma A431 cell line) [68]. Despite the fact that gigantoxin 4 and gigantoxin 5 are homological to gigantoxin Ι, no conclusion on their potential biological activity might be deduced from this fact.

*Acrorhagin homologs*. Although the presence of specialized aggressive organs named acrorhagi remains controversial in *A. viridis*, analyses of RNA-Seq data provides evidence for the production of four candidate toxins belonging to acrorhagin 1 and acrorhagin 2 subtypes [19]. Founder members of this class have been isolated from the acrorhagi of the sea anemone *Actinia equina* and have been reported to induce mortality when injected on crabs with LD_50_ values corresponding to 520 and 80 μg/kg for acrorhagin 1 and acrorhagin 2, respectively [69]. Computational tools for sequence and relation analysis fail to identify homologues superfamily and consistently they do not show significant sequence similarity (lower than 30% identity) with any toxins from other sources. Despite the fact that biological activity and functions have not yet been determined for this group of proteins, they may contribute to the articulated sea anemone venom assemblage.

All candidate toxins were found only on transcript level and their presence in venom needs to be validated in the future. Because the toxin expression profiles may vary greatly even inside single species, massive proteomics studies of venoms from different *A. viridis* specimens need to be carried out.

## 4. From Bioprospecting to Translational Research

Cnidarians possess exciting strategies for survival, which includes an articulated and finely defined toxin arsenal. Because of neurotoxicity and cytotoxicity of the sea anemone peptides, they represent promising putative pharmacological agents for translational research and biomedical applications. Moreover, different lines of evidences have defined cnidarian venom as an experimental tool for cell physiology [70]. The two *A. viridis* non-canonical Type 5 KTx represent interesting candidates for immune system targeting. Because the K_V_1.3 channel is responsible for the activity human effector memory T cells, the pharmacological application of recombinant or sinthetic Type 5 KTx may take place in the treatment of autoimmune diseases mediated by T cells as already described for the ShK toxin from the sea anemone *S. helianthus* [71,72]. Thus, blockers of K_V_1.3 channels show interesting application for the treatment of type 1 diabetes mellitus, rheumatoid arthritis and multiple sclerosis. BDS-1 has been pointed out as a promising potential tool for targeting Kv3.4 subunit, which has been implicated in CNS disorders as Parkinson’s and Alzheimer’s disease [73,74]. Several studies have reported the use of such an antagonist as a pharmacological tool to assign functional roles to channels in CNS neurons [74,75,76]. Effects were reported on several channels (K_V_3.4, K_V_3.1, K_V_3.2, and Na_V_1.7), thus instilling doubts on its future practical applications. It is noteworthy to specify that these reports take the advances of the use of purified sea anemone extracts, consisting of the BDS variants (1–14) which may display different specificity. Therefore, the development of new peptides with restricted activity by means of recombinant BDSs may allow the selective targeting of these channels.

Although several *A. viridis* toxins represent interesting candidates for biomedical applications, pharmacological investigations are still mandatory to reveal potency and selectivity of other *A. viridis* toxins, including those that have been only detected on the transcriptional level. High-throughput activity-testing systems are required to be developed to accelerate this process, thus allowing them to reach a different phase of clinical trials.

## Figures and Tables

**Table 1:** Transcriptome datasets of *A. viridis* available at NCBI.

Accession	Experiment Title	Platform	Submitter	Amount
ERX1926108 ERX1926107 ERX1926106 ERX1926105	Study of mitogenome and corresponding transcriptome of sea anemones	Ion Torrent PGM and Sanger technology	The Arctic University of Norway (UiT)	unspecified
SRX3049371 SRX3049370 SRX3049369 SRX3049368 SRX3049367 SRX3049366 SRX3049365 SRX3049364 SRX3049363	Small RNA sequencing of *Anemonia viridis*	AB 5500Xl Genetic Analyzer	Urbarova et al., 2018	2.9 × 10^3^ Mb
SRX699624	*Anemonia viridis* Transcriptome or Gene expression	Illumina HiSeq 2000	University of Haifa	5.4 × 10^3^ Mb
Under different accession	Symbiotic sea anemone *A. viridis* cDNA library	ABI-3730 Genetic Analyze (Sanger Technology)	Sabourault et al., 2010	39,939 ESTs
SRX971460	Tissue specific transcriptomes of the emerging model organism *Anemonia sulcata*	Illumina HiSeq 1500	The Ohio State University	8.8 × 10^3^ Mb
SRX971488	Tissue specific transcriptomes	Illumina HiSeq 2000	The Ohio State University	33.9 × 10^3^ Mb

## References

[1] Molinski T.F., Dalisay D.S., Lievens S.L., Saludes J.P. (2009). Drug development from marine natural products. Nat. Rev. Drug Discov..

[2] Glaser K.B., Mayer A.M.S. (2009). A renaissance in marine pharmacology: from preclinical curiosity to clinical reality. Biochem. Pharmacol..

[3] Rocha J., Peixe L., Gomes N.C.M., Calado R. (2011). Cnidarians as a source of new marine bioactive compounds—An overview of the last decade and future steps for bioprospecting. Mar. Drugs.

[4] Chen J.-Y., Oliveri P., Gao F., Dornbos S.Q., Li C.-W., Bottjer D.J., Davidson E.H. (2002). Precambrian animal life: probable developmental and adult cnidarian forms from Southwest China. Dev. Biol..

[5] Ruppert E.E., Barnes R.D. (1994). Invertebrate Zoology.

[6] Beress L. (1982). Biologically active compounds from coelenterates. Pure Appl. Chem..

[7] Nicosia A., Maggio T., Mazzola S., Gianguzza F., Cuttitta A., Costa S. (2014). Characterization of small HSPs from *Anemonia viridis* reveals insights into molecular evolution of alpha crystallin genes among cnidarians. PLoS ONE.

[8] Nicosia A., Maggio T., Mazzola S., Cuttitta A. (2013). Evidence of accelerated evolution and ectodermal-specific expression of presumptive BDS toxin cDNAs from *Anemonia viridis*. Mar. Drugs.

[9] Moran Y., Weinberger H., Sullivan J.C., Reitzel A.M., Finnerty J.R., Gurevitz M. (2008). Concerted evolution of sea anemone neurotoxin genes is revealed through analysis of the *Nematostella vectensis* genome. Mol. Biol. Evol..

[10] Moran Y., Weinberger H., Lazarus N., Gur M., Kahn R., Gordon D., Gurevitz M. (2009). Fusion and retrotransposition events in the evolution of the sea anemone *Anemonia viridis* neurotoxin genes. J. Mol. Evol..

[11] Castañeda O., Harvey A.L. (2009). Discovery and characterization of cnidarian peptide toxins that affect neuronal potassium ion channels. Toxicon.

[12] Nicosia A., Maggio T., Costa S., Salamone M., Tagliavia M., Mazzola S., Gianguzza F., Cuttitta A. (2016). Maintenance of a Protein Structure in the Dynamic Evolution of TIMPs over 600 Million Years. Genome Biol. Evol..

[13] Bulati M., Longo A., Masullo T., Vlah S., Bennici C., Bonura A., Salamone M., Tagliavia M., Nicosia A., Mazzola S. (2016). Partially Purified Extracts of Sea Anemone *Anemonia viridis* Affect the Growth and Viability of Selected Tumour Cell Lines. Biomed Res. Int..

[14] Cuttitta A., Ragusa M.A., Costa S., Bennici C., Colombo P., Mazzola S., Gianguzza F., Nicosia A. (2017). Evolutionary conserved mechanisms pervade structure and transcriptional modulation of allograft inflammatory factor-1 from sea anemone *Anemonia viridis*. Fish Shellfish Immunol..

[15] Nicosia A., Bennici C., Biondo G., Costa S., Di Natale M., Masullo T., Monastero C., Ragusa M.A., Tagliavia M., Cuttitta A. (2018). Characterization of Translationally Controlled Tumour Protein from the Sea Anemone *Anemonia viridis* and Transcriptome Wide Identification of Cnidarian Homologues. Genes.

[16] Frazão B., Vasconcelos V., Antunes A. (2012). Sea anemone (Cnidaria, Anthozoa, Actiniaria) toxins: An overview. Mar. Drugs.

[17] Kozlov S., Grishin E. (2011). The mining of toxin-like polypeptides from EST database by single residue distribution analysis. BMC Genom..

[18] Kozlov S., Grishin E. (2012). Convenient nomenclature of cysteine-rich polypeptide toxins from sea anemones. Peptides.

[19] Macrander J., Broe M., Daly M. (2016). Tissue-Specific Venom Composition and Differential Gene Expression in Sea Anemones. Genome Biol. Evol..

[20] Sabourault C., Ganot P., Deleury E., Allemand D., Furla P. (2009). Comprehensive EST analysis of the symbiotic sea anemone, *Anemonia viridis*. BMC Genom..

[21] Urbarova I., Patel H., Forêt S., Karlsen B.O., Jørgensen T.E., Hall-Spencer J.M., Johansen S.D. (2018). Elucidating the Small Regulatory RNA Repertoire of the Sea Anemone *Anemonia viridis* Based on Whole Genome and Small RNA Sequencing. Genome Biol. Evol..

[22] Martens L., Hermjakob H., Jones P., Adamski M., Taylor C., States D., Gevaert K., Vandekerckhove J., Apweiler R. (2005). PRIDE: The proteomics identifications database. Proteomics.

[23] Farrah T., Deutsch E.W., Kreisberg R., Sun Z., Campbell D.S., Mendoza L., Kusebauch U., Brusniak M.-Y., Hüttenhain R., Schiess R. (2012). PASSEL: The PeptideAtlas SRMexperiment library. Proteomics.

[24] Wilhelm M., Schlegl J., Hahne H., Gholami A.M., Lieberenz M., Savitski M.M., Ziegler E., Butzmann L., Gessulat S., Marx H. (2014). Mass-spectrometry-based draft of the human proteome. Nature.

[25] Martens L., Vizcaíno J.A. (2017). A Golden Age for Working with Public Proteomics Data. Trends Biochem. Sci..

[26] Salamone M., Nicosia A., Bennici C., Quatrini P., Catania V., Mazzola S., Ghersi G., Cuttitta A. (2015). Comprehensive Analysis of a *Vibrio parahaemolyticus* Strain Extracellular Serine Protease VpSP37. PLoS ONE.

[27] Himaya S.W.A., Lewis R.J. (2018). Venomics-Accelerated Cone Snail Venom Peptide Discovery. Int. J. Mol. Sci..

[28] Salgado V.L., Kem W.R. (1992). Actions of three structurally distinct sea anemone toxins on crustacean and insect sodium channels. Toxicon.

[29] Norton R.S. (1991). Structure and structure-function relationships of sea anemone proteins that interact with the sodium channel. Toxicon.

[30] Molgó J., Mallart A. (1985). Effects of *Anemonia sulcata* toxin II on presynaptic currents and evoked transmitter release at neuromuscular junctions of the mouse. Pflugers Arch..

[31] Schweitz H., Vincent J.P., Barhanin J., Frelin C., Linden G., Hugues M., Lazdunski M. (1981). Purification and pharmacological properties of eight sea anemone toxins from *Anemonia sulcata*, *Anthopleura xanthogrammica*, *Stoichactis giganteus*, and *Actinodendron plumosum*. Biochemistry.

[32] Warashina A., Ogura T., Fujita S. (1988). Binding properties of sea anemone toxins to sodium channels in the crayfish giant axon. Comp. Biochem. Physiol. C, Comp. Pharmacol. Toxicol..

[33] Wunderer G., Fritz H., Wachter E., Machleidt W. (1976). Amino-acid sequence of a coelenterate toxin: Toxin II from *Anemonia sulcata*. Eur. J. Biochem..

[34] Mikov A.N., Kozlov S.A. (2015). Structural Features of Cysteine-Stabilized Polypeptides from Sea Anemones Venoms. Bioorg. Khim..

[35] Hartung K., Rathmayer W. (1985). *Anemonia sulcata* toxins modify activation and inactivation of Na+ currents in a crayfish neurone. Pflugers Arch..

[36] Widmer H., Wagner G., Schweitz H., Lazdunski M., Wüthrich K. (1988). The secondary structure of the toxin ATX Ia from *Anemonia sulcata* in aqueous solution determined on the basis of complete sequence-specific 1H-NMR assignments. Eur. J. Biochem..

[37] Widmer H., Billeter M., Wüthrich K. (1989). Three-dimensional structure of the neurotoxin ATX Ia from *Anemonia sulcata* in aqueous solution determined by nuclear magnetic resonance spectroscopy. Proteins.

[38] Wunderer G. (1978). The disulfide bridges of toxin II from *Anemonia sulcata*. Hoppe-Seyler’s Z. Physiol. Chem..

[39] Chahine M., Plante E., Kallen R.G. (1996). Sea anemone toxin (ATX II) modulation of heart and skeletal muscle sodium channel alpha-subunits expressed in tsA201 cells. J. Membr. Biol..

[40] Scheffler J.J., Tsugita A., Linden G., Schweitz H., Lazdunski M. (1982). The amino acid sequence of toxin V from *Anemonia sulcata*. Biochem. Biophys. Res. Commun..

[41] Martinez G., Kopeyan C. (1977). Toxin III from *Anemonia sulcata*: Primary structure. FEBS Lett..

[42] Moran Y., Kahn R., Cohen L., Gur M., Karbat I., Gordon D., Gurevitz M. (2007). Molecular analysis of the sea anemone toxin Av3 reveals selectivity to insects and demonstrates the heterogeneity of receptor site-3 on voltage-gated Na+ channels. Biochem. J..

[43] Notredame C., Higgins D.G., Heringa J. (2000). T-Coffee: A novel method for fast and accurate multiple sequence alignment. J. Mol. Biol..

[44] Osmakov D.I., Kozlov S.A., Andreev Y.A., Koshelev S.G., Sanamyan N.P., Sanamyan K.E., Dyachenko I.A., Bondarenko D.A., Murashev A.N., Mineev K.S. (2013). Sea Anemone Peptide with Uncommon β-Hairpin Structure Inhibits Acid-sensing Ion Channel 3 (ASIC3) and Reveals Analgesic Activity. J. Biol. Chem..

[45] Honma T., Hasegawa Y., Ishida M., Nagai H., Nagashima Y., Shiomi K. (2005). Isolation and molecular cloning of novel peptide toxins from the sea anemone *Antheopsis maculata*. Toxicon.

[46] Honma T., Shiomi K. (2006). Peptide toxins in sea anemones: Structural and functional aspects. Mar. Biotechnol..

[47] Honma T., Kawahata S., Ishida M., Nagai H., Nagashima Y., Shiomi K. (2008). Novel peptide toxins from the sea anemone *Stichodactyla haddoni*. Peptides.

[48] Tudor J.E., Pallaghy P.K., Pennington M.W., Norton R.S. (1996). Solution structure of ShK toxin, a novel potassium channel inhibitor from a sea anemone. Nat. Struct. Biol..

[49] Pennington M.W., Kem W.R., Mahnir V.M., Byrnes M.E., Zaydenberg I., Khaytin I., Krafte D.S., Hill R., Kaumaya P.T.P., Hodges R.S. (1995). Identification of essential residues in the potassium channel inhibitor ShK toxin: Analysis of monosubstituted analogs. Peptides: Chemistry, Structure and Biology.

[50] Schweitz H., Bruhn T., Guillemare E., Moinier D., Lancelin J.-M., Béress L., Lazdunski M. (1995). Kalicludines and Kaliseptine Two different classes of sea anemone toxins for voltage sensitive K+ channels. J. Biol. Chem..

[51] Petersen T.N., Brunak S., von Heijne G., Nielsen H. (2011). SignalP 4.0: Discriminating signal peptides from transmembrane regions. Nat. Methods.

[52] Orts D.J.B., Peigneur S., Madio B., Cassoli J.S., Montandon G.G., Pimenta A.M.C., Bicudo J.E.P.W., Freitas J.C., Zaharenko A.J., Tytgat J. (2013). Biochemical and electrophysiological characterization of two sea anemone type 1 potassium toxins from a geographically distant population of *Bunodosoma caissarum*. Mar. Drugs.

[53] Scheidig A.J., Hynes T.R., Pelletier L.A., Wells J.A., Kossiakoff A.A. (1997). Crystal structures of bovine chymotrypsin and trypsin complexed to the inhibitor domain of Alzheimer’s amyloid beta-protein precursor (APPI) and basic pancreatic trypsin inhibitor (BPTI): Engineering of inhibitors with altered specificities. Protein Sci..

[54] Zweckstetter M., Czisch M., Mayer U., Chu M.L., Zinth W., Timpl R., Holak T.A. (1996). Structure and multiple conformations of the kunitz-type domain from human type VI collagen alpha3(VI) chain in solution. Structure.

[55] Chen C., Hsu C.H., Su N.Y., Lin Y.C., Chiou S.H., Wu S.H. (2001). Solution structure of a Kunitz-type chymotrypsin inhibitor isolated from the elapid snake *Bungarus fasciatus*. J. Biol. Chem..

[56] Antuch W., Berndt K.D., Chávez M.A., Delfín J., Wüthrich K. (1993). The NMR solution structure of a Kunitz-type proteinase inhibitor from the sea anemone *Stichodactyla helianthus*. Eur. J. Biochem..

[57] Diochot S., Loret E., Bruhn T., Béress L., Lazdunski M. (2003). APETx1, a new toxin from the sea anemone *Anthopleura elegantissima*, blocks voltage-gated human ether-a-go-go-related gene potassium channels. Mol. Pharmacol..

[58] Diochot S., Baron A., Rash L.D., Deval E., Escoubas P., Scarzello S., Salinas M., Lazdunski M. (2004). A new sea anemone peptide, APETx2, inhibits ASIC3, a major acid-sensitive channel in sensory neurons. EMBO J..

[59] Blanchard M.G., Rash L.D., Kellenberger S. (2012). Inhibition of voltage-gated Na(+) currents in sensory neurones by the sea anemone toxin APETx2. Br. J. Pharmacol..

[60] Béress L., Doppelfeld I.S., Etschenberg E., Graf E., Henschen A., Zwick J. (1985). Polypeptides, Methods of Production and Their Uses as Antihypertensives. German Patent.

[61] Diochot S., Schweitz H., Béress L., Lazdunski M. (1998). Sea anemone peptides with a specific blocking activity against the fast inactivating potassium channel Kv3.4. J. Biol. Chem..

[62] Yeung S.Y.M., Thompson D., Wang Z., Fedida D., Robertson B. (2005). Modulation of Kv3 subfamily potassium currents by the sea anemone toxin BDS: Significance for CNS and biophysical studies. J. Neurosci..

[63] Abbott G.W., Butler M.H., Bendahhou S., Dalakas M.C., Ptacek L.J., Goldstein S.A. (2001). MiRP2 forms potassium channels in skeletal muscle with Kv3.4 and is associated with periodic paralysis. Cell.

[64] Liu P., Jo S., Bean B.P. (2012). Modulation of neuronal sodium channels by the sea anemone peptide BDS-I. J. Neurophysiol..

[65] Driscoll P.C., Gronenborn A.M., Beress L., Clore G.M. (1989). Determination of the three-dimensional solution structure of the antihypertensive and antiviral protein BDS-I from the sea anemone *Anemonia sulcata*: A study using nuclear magnetic resonance and hybrid distance geometry-dynamical simulated annealing. Biochemistry.

[66] Chagot B., Diochot S., Pimentel C., Lazdunski M., Darbon H. (2005). Solution structure of APETx1 from the sea anemone Anthopleura elegantissima: A new fold for an HERG toxin. Proteins.

[67] Chagot B., Escoubas P., Diochot S., Bernard C., Lazdunski M., Darbon H. (2005). Solution structure of APETx2, a specific peptide inhibitor of ASIC3 proton-gated channels. Protein Sci..

[68] Shiomi K., Honma T., Ide M., Nagashima Y., Ishida M., Chino M. (2003). An epidermal growth factor-like toxin and two sodium channel toxins from the sea anemone *Stichodactyla gigantea*. Toxicon.

[69] Honma T., Minagawa S., Nagai H., Ishida M., Nagashima Y., Shiomi K. (2005). Novel peptide toxins from acrorhagi, aggressive organs of the sea anemone *Actinia equina*. Toxicon.

[70] Morabito R., La Spada G., Crupi R., Esposito E., Marino A. (2015). Crude Venom from Nematocysts of the Jellyfish *Pelagia noctiluca* as a Tool to Study Cell Physiology. Cent. Nerv. Syst. Agents Med. Chem..

[71] Prentis P.J., Pavasovic A., Norton R.S. (2018). Sea Anemones: Quiet Achievers in the Field of Peptide Toxins. Toxins.

[72] Chi V., Pennington M.W., Norton R.S., Tarcha E.J., Londono L.M., Sims-Fahey B., Upadhyay S.K., Lakey J.T., Iadonato S., Wulff H. (2012). Development of a sea anemone toxin as an immunomodulator for therapy of autoimmune diseases. Toxicon.

[73] Baranauskas G., Tkatch T., Nagata K., Yeh J.Z., Surmeier D.J. (2003). Kv3.4 subunits enhance the repolarizing efficiency of Kv3.1 channels in fast-spiking neurons. Nat. Neurosci..

[74] Angulo E., Noe V., Casado V., Mallol J., Gomez-Isla T., Lluis C., Ferrer I., Ciudad C.J., Franco R. (2004). Up-regulation of the Kv3.4 potassium channel subunit in early stages of Alzheimer’s disease. J. Neurochem..

[75] Chabbert C., Chambard J.M., Sans A., Desmadryl G. (2001). Three types of depolarization-activated potassium currents in acutely isolated mouse vestibular neurons. J. Neurophysiol..

[76] Shevchenko T., Teruyama R., Armstrong W.E. (2004). High-threshold, Kv3-like potassium currents in magnocellular neurosecretory neurons and their role in spike repolarization. J. Neurophysiol..

